# Green Synthesis and Characterization of Zinc Oxide Nanoparticles Using Catharanthus roseus Extract: A Novel Approach

**DOI:** 10.7759/cureus.60407

**Published:** 2024-05-16

**Authors:** Sankari Malaiappan, Priyangha P T, Sankari Niveditha

**Affiliations:** 1 Department of Periodontics, Saveetha Dental College and Hospitals, Saveetha Institute of Medical and Technical Sciences, Saveetha University, Chennai, IND; 2 Department of Dentistry, Saveetha Dental College and Hospitals, Saveetha Institute of Medical and Technical Sciences, Saveetha University, Chennai, IND

**Keywords:** anti-inflammatory, anti-oxidant, zinc oxide nanoparticles, green synthesis, catharanthus roseus

## Abstract

Background: Nanotechnology enables precise manipulation of matter at the molecular level, with nanoparticles offering diverse applications in medicine and beyond. Green synthesis methods, utilizing natural sources like plant extracts, are favored for their eco-friendliness. Zinc oxide (ZnO) nanoparticles are recognized for their ability to combat microbes and reduce inflammation, which holds promise for biomedical applications. *Catharanthus roseus*, renowned for its medicinal properties, warrants further exploration in oral health management due to its anti-inflammatory and antioxidant attributes.

Aim: The current study aimed to synthesize* Catharanthus roseus*-mediated ZnO nanoparticles and to evaluate their anti-inflammatory and antioxidant activity.

Materials and methods: *Catharanthus roseus* powder (1 g) was dissolved in distilled water (100 ml), heated at 60°C for 15-20 minutes, and filtered to obtain 20 ml extract. ZnO nanoparticles were synthesized by adding 0.594 g ZnO powder to 50 ml water, mixed with plant extract, and stirred for 72 hours, and the resulting solution was centrifuged. Nanoparticles were collected and analyzed for Fourier-transform infrared spectroscopy (FTIR) using Bruker’s Alpha II FTIR spectrometer (Bruker, Billerica, Massachusetts, United States), antioxidant, and anti-inflammatory activities.

Results: FTIR analysis revealed characteristic peaks indicative of functional groups present in *Catharanthus roseus*-mediated ZnO nanoparticles, including O-H, N-O, C-O, C=C, and C≡C-H. Anti-inflammatory activity evaluation showed inhibition ranging from 48% to 89%, with the maximum inhibition at 50 μL concentration. Similarly, antioxidant activity ranged from 62% to 88%, with the maximum inhibition also seen at 50 μL concentration.

Conclusion: Both assays effectively showcased the superior anti-inflammatory and antioxidant activity of the *Catharanthus roseus*-incorporated ZnO nanoparticles extract compared to the control. This suggests their potential as a viable therapeutic agent for further evaluation.

## Introduction

Nanotechnology encompasses manipulating matter at the atomic or molecular level, with nanoparticles serving as pivotal components typically ranging from 1 to 100 nanometers in size [1]. These particles exhibit distinctive physical, chemical, and biological properties due to their minuscule dimensions, which can be tailored for diverse applications including drug delivery, catalysis, and imaging [2]. The precise control afforded by nanoparticles fosters the creation of innovative solutions across various sectors, from medicine to environmental remediation, promising substantial advancements in numerous industries and scientific domains [3]. Continued interest in this field, particularly within nanomedicine, is fueled by the potential to refine existing products into novel versions with superior properties. Nanoparticle synthesis encompasses various physical and chemical processes [4].

Despite their effectiveness, these synthesis methods have drawbacks, often involving the use of toxic chemicals that contribute to environmental pollution and pose challenges for hazardous waste disposal. While chemical synthesis techniques may prove successful at the laboratory scale, scaling up production to industrial levels presents obstacles related to reproducibility, cost-effectiveness, and process optimization. Addressing these limitations, the ongoing research was aimed at developing greener and more sustainable synthesis approaches for nanoparticles, alongside efforts to enhance the scalability, reproducibility, and efficiency of existing chemical methods.

Hence, contemporary approaches to nanoparticle synthesis prioritize eco-friendly methods that bypass hazardous chemicals and extreme conditions [5]. These techniques often leverage natural sources like plant extracts (such as green tea, neem, or aloe vera) or microorganisms, alongside other environmentally sound materials serving as reducing agents, stabilizers, or templates for nanoparticle synthesis [6].

Zinc oxide (ZnO) nanoparticles have garnered substantial interest due to their affordability, safety, and compatibility with biological systems [7]. They exhibit noteworthy antimicrobial properties capable of engaging bacterial surfaces with distinct bactericidal mechanisms and are effective against a wide range of microorganisms [8]. Zinc also exhibits greater chemical stability when compared to other physiological metals [9].

Herbal remedies, known for their minimal side effects, have historically played a pivotal role in traditional and alternative medicine [10]. Among them, *Catharanthus roseus*, a member of the Apocynaceae family commonly known as periwinkle or Madagascar-periwinkle, stands out for its medicinal significance [11]. Abundant in alkaloids like vincristine and vinblastine, *Catharanthus roseus* boasts a rich history of therapeutic applications in managing conditions such as diabetes, wounds, scurvy, hypertension, and malaria [12].

Despite its medicinal prowess, scant literature explores the use of *Catharanthus roseus* extracts or its constituents in oral care products thus underscoring the need for further investigation. Given the plant's recognized benefits and potential therapeutic properties, particularly its anti-inflammatory and antioxidant attributes, its exploration in oral health management becomes imperative. Thus, the present study aimed to synthesize and evaluate the anti-inflammatory and antioxidant activity of *Catharanthus roseus*-mediated ZnO nanoparticles.

## Materials and methods

Preparation of plant extract

**Figure 1:**
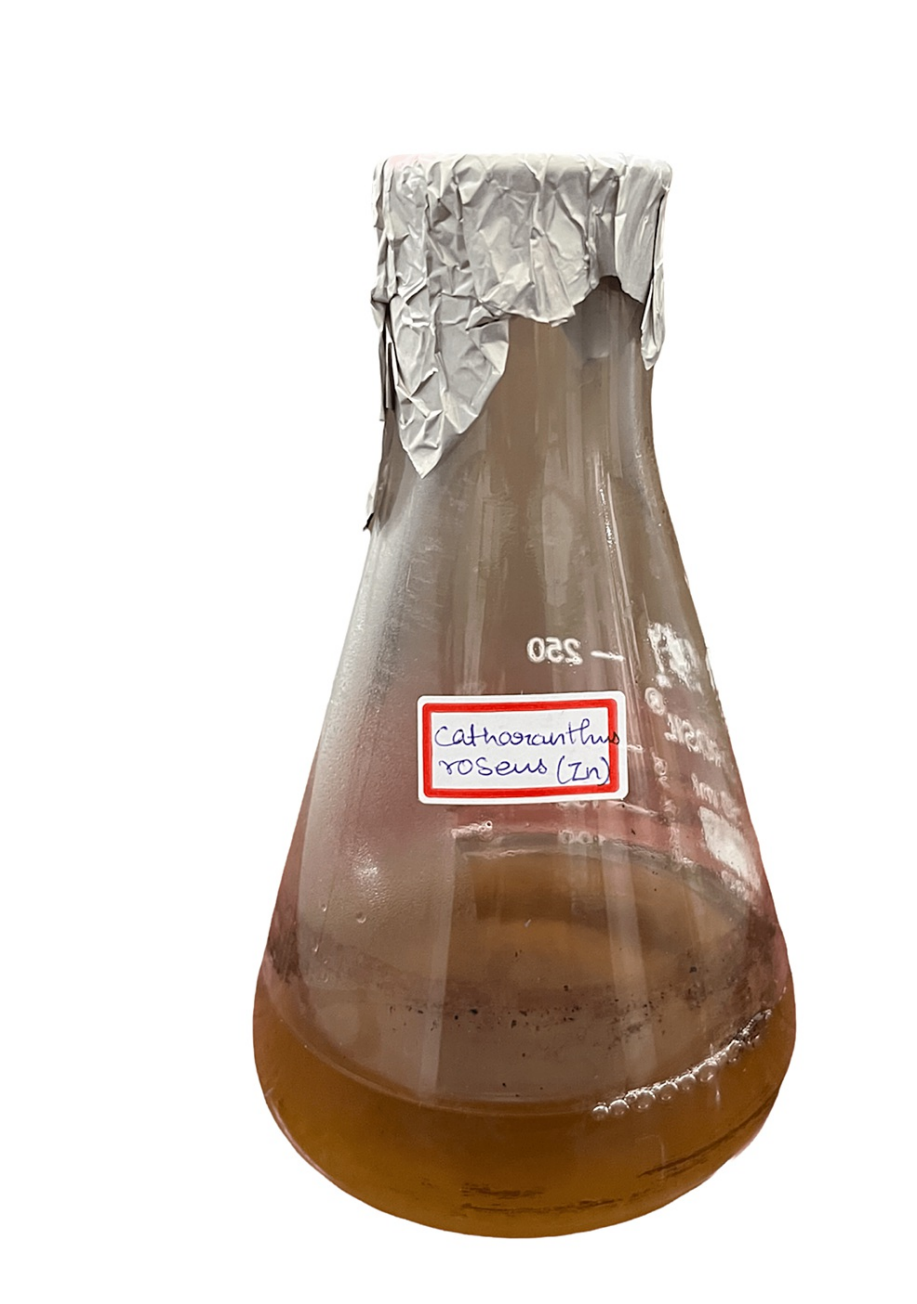
Preparation of the plant extract

About 1 g of freshly collected and powdered *Catharanthus roseus* was dissolved in distilled water (100 ml) followed by heating for 15 to 20 minutes at 60°C using a heating mantle. Subsequently, the extract was filtered using Whatman filter paper to obtain 20 ml of filtered extract which was later utilized for green synthesis as illustrated in Figure 1.

Synthesis of ZnO nanoparticles

To 50 ml of distilled water, 0.594 g of ZnO powder and 20 ml of the prepared plant extract were added, and the mixture was stirred and shaken at 900 rpm for 72 hours. After synthesis, the extract was collected and distributed into five centrifuge tubes each containing 2 ml and was centrifuged for 10 minutes. The sedimented nanoparticles were then collected from the tubes and transferred into a beaker. Nanoparticles were extracted from the tubes using filler and scrapers. Subsequently, the obtained nanoparticles were subjected to Fourier-transform infrared spectroscopy (FTIR), antioxidant, and anti-inflammatory activity analysis.

FTIR analysis

The investigation into the interaction between* Catharanthus roseus* and ZnO nanoparticles was carried out using a Bruker’s Alpha II FTIR spectrometer(Bruker, Billerica, Massachusetts, United States) and the spectra were recorded using dry films. To create these films, the formulated substance containing *Catharanthus roseus* ZnO nanoparticles was poured into a polystyrene Petri plate and was dried for three days.

Anti-inflammatory activity

*Bovine Serum Albumin (BSA)* *Assay *

BSA assay constitutes approximately 60% of all proteins in animal serum and undergoes denaturation upon heating, triggering an inflammatory response associated with type 3 hypersensitivity reactions. In this assay, 0.05 ml of *Catharanthus roseus*-incorporated nanoparticles were added to BSA fraction (2 ml). Different concentrations ranging from 10 μl to 50 μl were distributed. The solution was later mixed with 1 N hydrochloric acid at a pH of 6.8, incubated at ambient temperature for 20 minutes, and allowed to cool down. Spectrophotometric analysis of absorbance values set at 660 nm was used to determine the absorbance. The standard control was diclofenac sodium, acknowledged for its anti-inflammatory properties.

% protein denaturation inhibition: = {Absorbance of control - Absorbance of sample} x 100 / Absorbance of control.

Egg Albumin (EA) Denaturation Assay

A mixture was prepared by combining 0.2 ml of egg albumin with 2.8 ml of phosphate-buffered saline (PBS) to create a solution of 5 ml. Various concentrations of the synthesized nanoparticle extracts ranging from 10 µL to 50 µL were prepared and kept. Diclofenac was employed as the control. The solutions were then heated for 15 minutes at 37°C and permitted to cool to room temperature. Absorption measurements were taken at 660 nm.

Antioxidant activity

2,2-Diphenyl-1-Picrylhydrazyl​​​ (DPPH) Assay

Five test tubes were taken, and 0.1 mM DPPH solution and 50% methanol solution (buffer) were added to the test tubes followed by the addition of different concentrations of prepared nanoparticles extract and incubated in darkness for 30 minutes. Spectrophotometric analysis of absorbance values was conducted at 517 nm against the standard ascorbic acid.

% Minimum inhibitory concentration = (Absorbance of control - Absorbance of the sample) x 100/Absorbance of control.

*Hydrogen Peroxide *​​*(*​​​​​*H_2_O_2_) Assay*

The reagents used were 0.5 mL of ethylenediaminetetraacetic acid (EDTA), 1.0 mL of dimethyl sulfoxide, and 0.5 mL of ascorbic acid. About 1 mL of trichloroacetic acid (17.5%) served as the terminating agent for the reaction. Subsequently, 3 mL of Nash reagent and distilled water (1 L) were added followed by incubation for 15 minutes at ambient temperature, after which a yellow color was observed. The intensity and degree of inhibition were assessed at 412 nm, with ascorbic acid serving as the standard.

## Results

FTIR analysis

**Figure 2:**
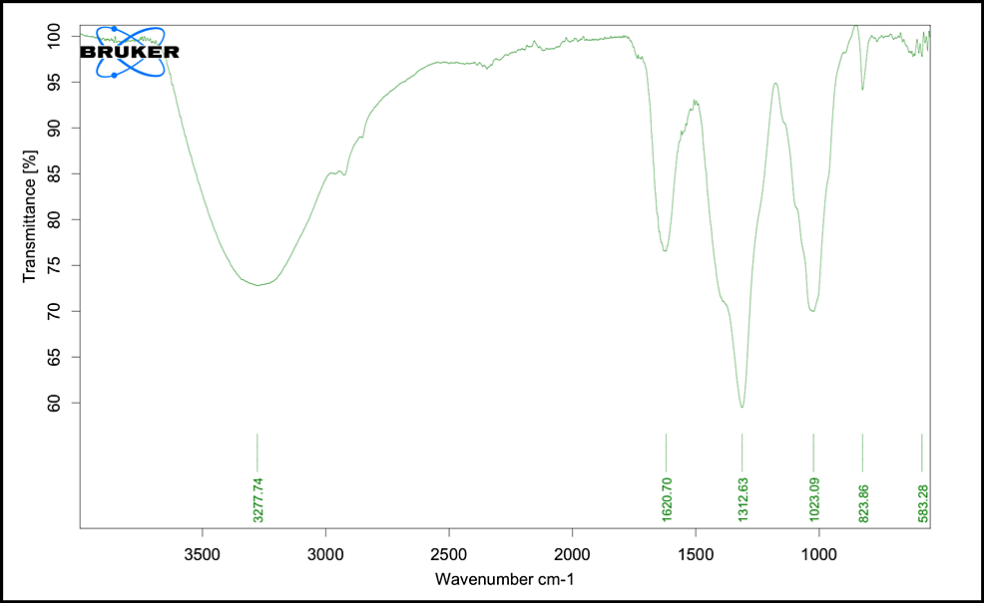
FTIR analysis with X-axis showing the wavenumber and Y-axis showing the transmittance of Catharanthus roseus-mediated zinc oxide nanoparticles FTIR: Fourier-transform infrared spectroscopy

FTIR results as depicted in Figure 2 provide insight into the chemical composition and functional groups present in *Catharanthus roseus*-mediated ZnO nanoparticles, indicating the involvement of organic molecules from the plant extract in the synthesis process and the potential role of these functional groups in stabilizing the nanoparticles. The FTIR results are tabulated in Table 1 correspondingly.

**Table 1:** The presence of various functional groups in Catharanthus roseus-mediated zinc oxide nanoparticles based on the peaks observed in its infrared (IR) spectrum

Peak (cm^-1^)	Functional group	Chemical description
3277.74	O-H stretch	Phenol groups (hydroxyl functional groups)
1620.70	N-O stretch	Nitro or nitroso functional groups
1312.63	N-O stretch	Nitro or nitroso functional groups
1023.09	C-O stretch	Alcohol, ether, or ester functional groups
823.86	C=C bend	Unsaturated hydrocarbons or aromatic compounds
583.28	C≡C-H peak	Alkyne groups (triple-bonded carbon atoms)

Assessment of anti-inflammatory activity

**Figure 3:**
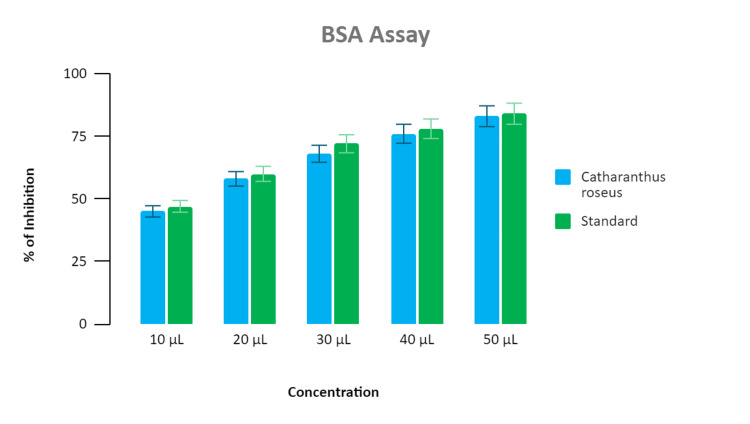
Bar graph depicting the anti-inflammatory activity of Catharanthus roseus (BSA assay) The graph represents an association between the concentration and percentage of inhibition shown by the standard (diclofenac) and *Catharanthus roseus-*mediated zinc oxide nanoparticle. The X-axis represents the concentration of extract added in microliter (μl), and the Y-axis represents the percentage of inhibition shown by the standard and *Catharanthus roseus* extract

**Figure 4:**
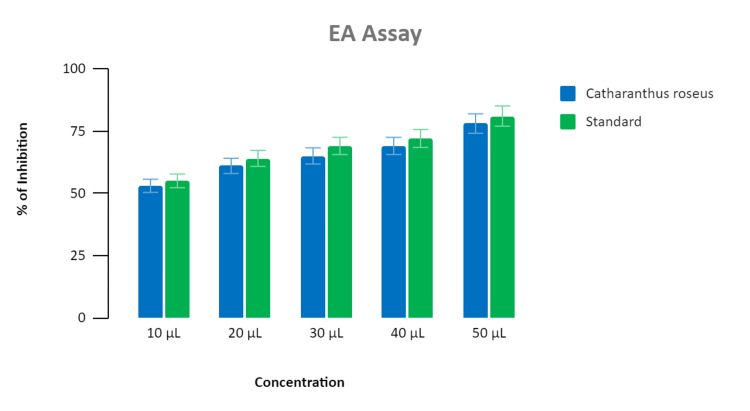
Bar graph depicting the anti-inflammatory activity of Catharanthus roseus (EA assay) The graph represents an association between the concentration and percentage of inhibition shown by the standard (diclofenac) and *Catharanthus roseus-*mediated zinc oxide nanoparticles. The X-axis represents the concentration of extract added in microliter (μl), and the Y-axis represents the percentage of inhibition shown by the standard and *Catharanthus roseus *extract

The anti-inflammatory activity was estimated for multiple intensities ranging from 10 μL to 50 μL. About 90% inhibition was proven for the standard (diclofenac) and the synthesized nanoparticles extract demonstrated inhibitions at 48%, 50%, 75%, 85%, and 89%, respectively. The highest level of anti-inflammatory activity of the synthesized nanoparticle extract was recorded at 50 μL, and it was found to be 89% inhibition in the BSA assay as depicted in Figure 3, and 88% inhibition in the EA assay as depicted in Figure 4.

Assessment of antioxidant activity

**Figure 5:**
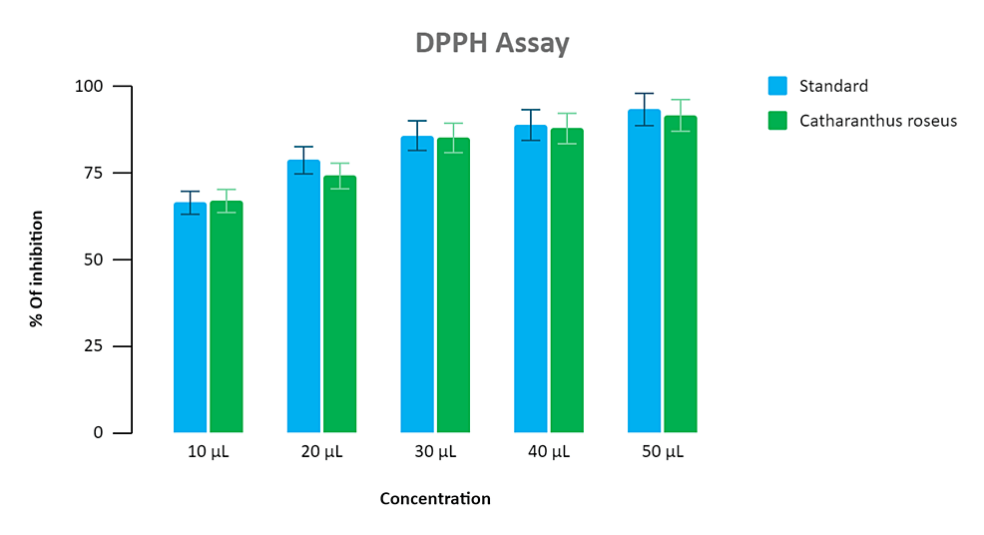
Bar graph depicting the antioxidant activity of Catharanthus roseus (DPPH assay) The graph represents an association between the concentration and percentage of inhibition shown by the standard and *Catharanthus roseus-*mediated zinc oxide nanoparticles. The X-axis represents the different concentration of standard (vitamin C)  and *Catharanthus roseus* extract added in microliters (μl), and the Y-axis represents the percentage of inhibition shown by the standard and *Catharanthus roseus* extract

**Figure 6:**
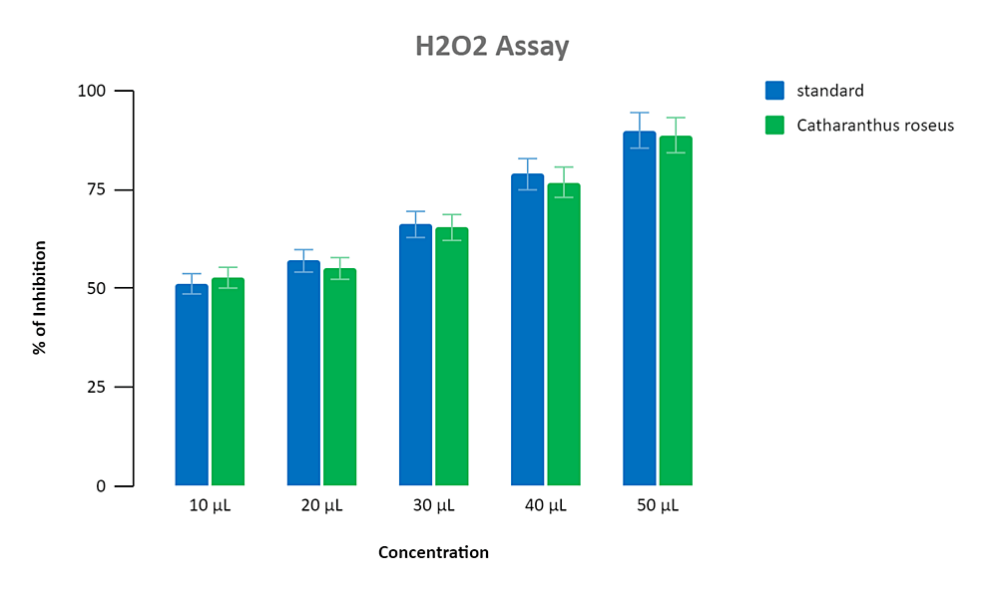
Bar graph depicting the antioxidant activity of Catharanthus roseus (H2O2 assay) The graph represents an association between the concentration and percentage of inhibition shown by the standard and *Catharanthus roseus-*mediated zinc oxide nanoparticles. The X-axis represents the different concentration of standard (vitamin C)  and *Catharanthus roseus* extract added in microliters (μl), and the Y-axis represents the percentage of inhibition shown by the standard and *Catharanthus roseus *extract

Antioxidant activity was assessed for multiple strengths (10 μL to 50 μL) of the synthesized nanoparticle extract. About 90% inhibition in the DPPH assay and 87% inhibition in the H₂O₂ assay was noted for the standard (ascorbic acid). While for the synthesized nanoparticles extract, the inhibition at different concentrations were 62%, 75%, 77%, 79%, and 88%, respectively. The highest antioxidant activity for both the DPPH assay and H₂O₂ assay was recorded at a concentration of 50 μL and was found to be 88% as shown in Figure 5 and Figure 6.

## Discussion

The emergence of green synthesis in nanoparticle production signifies a transformative shift, offering not only eco-friendly attributes but also a multitude of other benefits [13]. Utilizing the reducing and stabilizing properties of natural sources like plant extracts, microorganisms, and biomolecules, this method enables the creation of nanoparticles under mild reaction conditions. Moreover, the inherent biocompatibility of nanoparticles synthesized through green methods renders them highly suitable for biomedical applications. Beyond its technical advantages, green synthesis aligns with broader sustainability objectives, promoting cleaner production practices and fostering innovation in line with sustainable development goals.

Nanotechnology, a multidisciplinary field intersecting physics, chemistry, biology, and engineering, holds immense potential for revolutionizing various industries and societal sectors. In the realm of medicine, nanotechnology offers more efficient treatments with reduced side effects while also contributing significantly to environmental sustainability [14]. Its applications span from pollution remediation and water purification to energy storage and conservation. Despite its promising potential, nanotechnology poses safety considerations that necessitate careful and responsible development. Therefore, our current study was focused on the green synthesis of nanoparticles to mitigate environmental risks.

*Catharanthus roseus*, a medicinal plant with a rich history of traditional uses and modern applications, has garnered considerable attention for its numerous advantages. Studies have demonstrated the plant's efficacy with phytochemicals derived from *Catharanthus roseus *showing significant health benefits. Notably, compounds like vinblastine and vincristine extracted from the plant exhibit anticancer properties, while newly discovered indole alkaloids such as catharoseumine have demonstrated promising results in inhibiting human cancer cell lines in vitro [15]. A study on the pharmacological activity of *Catharanthus roseus *revealed the presence of 130 alkaloids in the plant, which exhibit potential effects against leukemia, diabetes, and aid in wound healing [16].

The objective of our study was to assess the in vitro characteristics of the formulated *Catharanthus roseus*-mediated ZnO nanoparticle. In the FTIR analysis, peaks were detected within a range from 583.28 cm⁻¹ to 3277.74 cm⁻¹, corresponding to stretching modes of hydroxyl groups and carbon-hydrogen bonds, respectively. These results correlate with a study on the potent antimicrobial properties of green ZnO nanoparticles derived from *Catharanthus roseus*, which likewise detected comparable peaks falling within the range of 500-4000 cm⁻¹ [17].

In our study, the anti-inflammatory and antioxidant properties of the formulation were studied and the shift in color from yellow to brownish-black suggested the replacement of metallic zinc (Zn+) ions. These findings affirm the capacity of ZnO nanoparticles synthesized through* Catharanthus roseus* to mitigate inflammation and alleviate oxidative stress. These results align with a study investigating the eco-friendly production of silver nanoparticles utilizing extracts from *Catharanthus roseus *flowers, which also demonstrated significant antioxidant and antimicrobial activities against Gram-negative *Escherichia coli* [18]. In a separate study, researchers investigated the phytochemistry, cytotoxicity, and antiviral activity of *Catharanthus roseus*, concluding that the plant extract holds promising potential as an anti-HSV-1 agent [19].

Despite the numerous methods available for nanoparticle synthesis, green synthesis is preferred due to its simplicity, cost-effectiveness, and its biocompatible nature. Nowadays with all these newer technologies, there is a growing focus on environmentally friendly methods for nanoparticle production. Recent reports have detailed the production of ZnO nanoparticles using various green approaches [20]. The bioactive compounds present in *Catharanthus roseus*, such as alkaloids, flavonoids, terpenoids, and phenolic compounds, act as reducing agents or stabilizers during nanoparticle synthesis. These green-synthesized nanoparticles, due to their unique properties and reduced environmental impact, render them valuable for diverse applications [21]. Thus in our study, *Catharanthus roseus* was employed to produce ZnO nanoparticles.

Exploring the realm of nanotechnology in conjunction with traditional herbal medicine opens doors to promising advancements in healthcare. By harnessing nanoscale drug delivery systems, herbal remedies stand poised to exhibit heightened biological efficacy while circumventing constraints often encountered with synthetic drugs. The fusion of herbal medicines with nanoparticle-based delivery platforms not only augments disease management strategies. Furthermore, the integration of herbal nanoparticles holds immense potential in diverse fields, including preventive oral healthcare. From enhancing the longevity and resilience of dental prostheses to aiding in teeth implantation procedures, herbal nanoparticles shows multifaceted benefits in oral health. Embracing this innovative approach not only holds promise in combating oral diseases such as oral cancer but also fosters a holistic approach to oral well-being.

Strength and constraints

The primary benefit here is the utilization of the green synthesis method, coupled with the biocompatibility and low toxicity associated with *Catharanthus roseus*, rendering it an ideal candidate for biomedical applications. Although the ZnO nanoparticles mediated by *Catharanthus roseus* exhibit promise in terms of anti-inflammatory and antioxidant activity, further studies are required to evaluate their cytotoxic potential comprehensively. It is essential to further explore in terms of clinical experiments and to validate and expand upon the findings of this study.

## Conclusions

In conclusion, within the confines of this study, it is evident that the anti-inflammatory and antioxidant characteristics of the ZnO nanoparticle formulation facilitated by *Catharanthus roseus* were observed to be highly effective. These environmentally friendly synthesized nanoparticles present promising prospects as valuable supplements to existing dental treatments, meriting thorough investigation for their therapeutic roles in oral lesions. Moreover, to maximize their effectiveness, these nanoparticles synthesized through eco-friendly methods could be integrated into dental materials or be applied as coatings on suture materials. Additionally, they hold the potential for driving innovation in drug development, paving the way for the creation of novel therapeutics that boast heightened potency and reduced toxicity profiles. Further exploration into these avenues promises to revolutionize dental care and advance oral health management strategies.
